# Arabidopsis myosin XI sub-domains homologous to the yeast myo2p organelle inheritance sub-domain target subcellular structures in plant cells

**DOI:** 10.3389/fpls.2013.00407

**Published:** 2013-10-22

**Authors:** Amirali Sattarzadeh, Elmon Schmelzer, Maureen R. Hanson

**Affiliations:** ^1^Department of Molecular Biology and Genetics, Cornell UniversityIthaca, NY, USA; ^2^Central Microscopy, Max-Planck-Institute for Plant Breeding ResearchCologne, Germany

**Keywords:** yeast myo2p, myosin V, transient expression, Golgi, mitochondria, vacuole, confocal microscopy, *Nicotiana benthamiana*

## Abstract

Myosin XI motor proteins transport plant organelles on the actin cytoskeleton. The Arabidopsis gene family that encodes myosin XI has 13 members, 12 of which have sub-domains within the tail region that are homologous to well-characterized cargo-binding domains in the yeast myosin V myo2p. Little is presently known about the cargo-binding domains of plant myosin XIs. Prior experiments in which most or all of the tail regions of myosin XIs have been fused to yellow fluorescent protein (YFP) and transiently expressed have often not resulted in fluorescent labeling of plant organelles. We identified 42 amino-acid regions within 12 Arabidopsis myosin XIs that are homologous to the yeast myo2p tail region known to be essential for vacuole and mitochondrial inheritance. A YFP fusion of the yeast region expressed in plants did not label tonoplasts or mitochondria. We investigated whether the homologous Arabidopsis regions, termed by us the “PAL” sub-domain, could associate with subcellular structures following transient expression of fusions with YFP in *Nicotiana benthamiana*. Seven YFP::PAL sub-domain fusions decorated Golgi and six were localized to mitochondria. In general, the myosin XI PAL sub-domains labeled organelles whose motility had previously been observed to be affected by mutagenesis or dominant negative assays with the respective myosins. Simultaneous transient expression of the PAL sub-domains of myosin XI-H, XI-I, and XI-K resulted in inhibition of movement of mitochondria and Golgi.

## Introduction

Myosins, molecular motors that move cargo along actin filaments, occur in all eukaryotic organisms. Based on sequence homology, the myosin super family can be divided into 37 classes (Richards and Cavalier-Smith, 2005; Foth et al., 2006). The myosins from higher plants fall within only two classes: class VIII and XI (Hodge and Cope, 2000; Berg et al., 2001). Plant myosins have the domain pattern that is characteristic for most of the myosins: (1) the extremely conserved N-terminal head (motor) domain, which binds to actin filaments and is responsible for force production via ATP hydrolysis (2) the neck (lever arm) domain, including distinctive repeat motifs called IQ repeats and (3) the C-terminal tail domain that facilitates cargo binding. Like most myosins, the tails of plant myosins are highly divergent domains that vary widely in length and in sequence. In addition, the tails of plant myosins have coiled-coil-forming sequences that allow the molecules to dimerize, producing two-headed molecules. In general, the tail domains of myosins are believed to be largely responsible for class-specific functions.

ATM1, which is a class VIII myosin, was the first plant myosin to be characterized, and was implicated in *Arabidopsis thaliana* cell wall formation (Reichelt et al., 1999). Recently, class VIII myosins were shown to be involved in plasmodesmata- mediated intracellular trafficking and endocytosis (Avisar et al., 2008a; Golomb et al., 2008; Sattarzadeh et al., 2008). In contrast to class VIII myosins, class XI myosins are similar to the well-studied myosin V proteins of fungi and animals. In Arabidopsis, a 13-member gene family encodes the myosin XIs (Reddy, 2001; Tominaga and Nakano, 2012). Analysis of insertional mutants of the Arabidopsis myosin XIs revealed considerable redundancy of function, as little effect on plant growth and organelle dynamics was detected (Ojangu et al., 2007; Peremyslov et al., 2008). Single and higher order mutants and RNA silencing have been used to probe functions of the myosin XI family (Ojangu et al., 2007; Prokhnevsky et al., 2008; Sparkes et al., 2008; Avisar et al., 2009). Incomplete myosins have been expressed, carrying putative cargo-binding domains but lacking the motor, so that if such proteins bind a large proportion of the available receptors on a cargo, there should be a dominant negative effect on movement of a particular organelle (Prokhnevsky et al., 2008; Sparkes et al., 2008; Avisar et al., 2009). Replacing the motor domain with fluorescent proteins has resulted in mixed success regarding labeling of particular organelles. Depending on the region of the myosin that has been expressed, sometimes minimal labeling of subcellular structures have been observed, with most fluorescence remaining in the cytoplasm (Reisen and Hanson, 2007; Avisar et al., 2008b, 2009, 2012). The particular portion of the myosin XI tail that has been used in such experiments has a strong effect on whether or not fluorescent vesicles and subcellular structures are observed.

**Figure 1:**
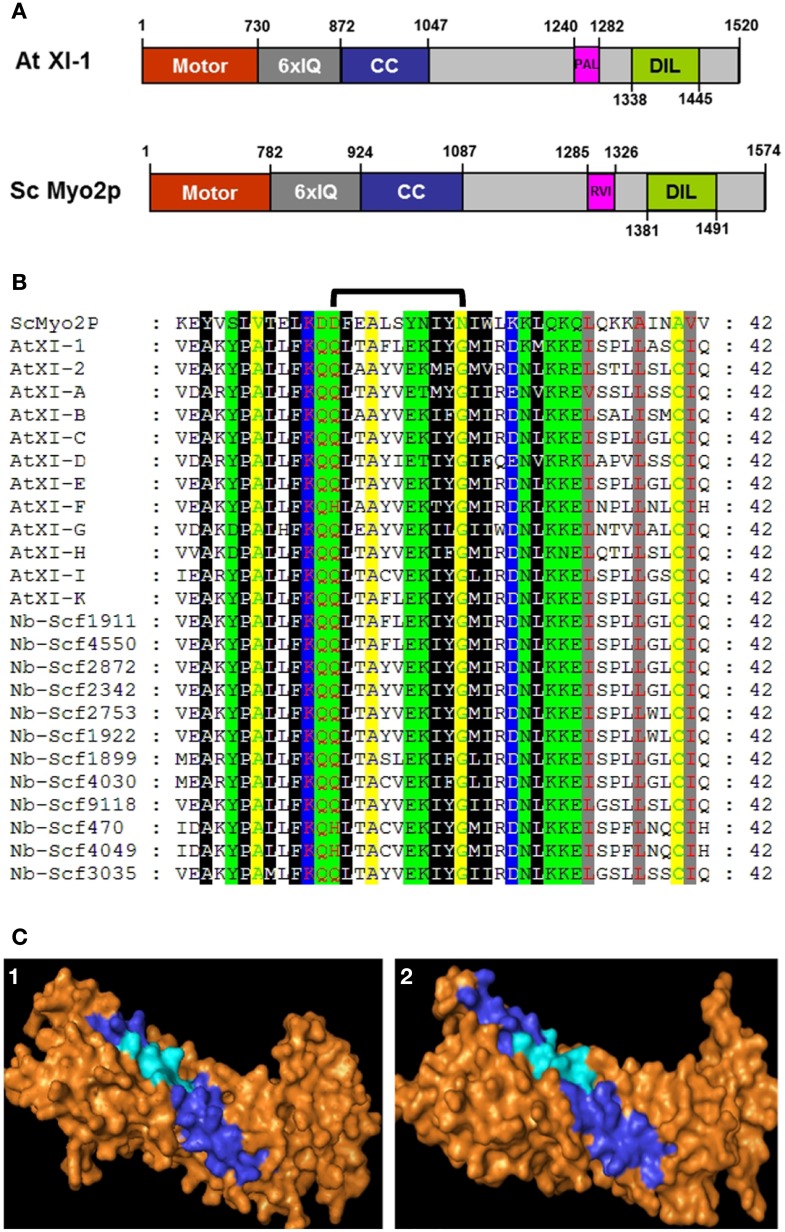
**Comparison of At XI-1 and yeast myo2p. (A)** Diagram of *A. thaliana* myosin At XI-1 and yeast myosin V ScMyo2p. Motor domain, IQ repeats, coiled-coil (CC) regions and domains in globular tail of both proteins are shown. The PAL sub-domain, the cargo binding site from At XI-1 identified as homologous to ScMyo2p vacuole binding site (signified with RVI), and DIL domains are shown. Drawn approximately to scale except for the motor domain. **(B)** Alignment of the PAL sub-domains from *A. thaliana* class and *N. benthamiana* XI myosins to the homologous domain in the yeast Sc Myo2p. The 11 a.a region critical for vacuole binding (Catlett et al., 2000) in ScMyo2p is indicated by a bracket. **(C)** Homology structural modeling of myosin XI globular tail based on similarity to the myosin V ScMyo2p. Predicted three-dimensional structures of the globular tail of *A. thaliana* class XI-K myosin is illustrated. (1) At XI-K, (2) ScMyo2p. Surface residues are shown in orange. The 11 amino acids critical for the vacuole binding site in ScMyo2p tail (Catlett et al., 2000) and the corresponding amino acids in PAL sub-domains are shown in cyan, while the entire 42-amino acid PAL domain is shown in dark blue.

Class V myosin tails such as yeast myo2p contain two functional domains which harbor vacuole- and mitochondrial-specific and secretory vesicle-specific regions (Pashkova et al., 2005, 2006; Fortsch et al., 2011). Regions homologous to the two domains exist in the Arabidopsis myosin XI family. Previously we have described the labeling of subcellular locations by YFP fusions to the DIL (dilute) domain (similar to the yeast secretory vesicle-specific domain) of 12 Arabidopsis myosin XIs (Sattarzadeh et al., 2011). Peroxisomes were labeled by YFP fusions to DIL domains of 8 different myosin XIs, three labeled Golgi, and others labeled the plasma membrane, endoplasmic reticulum (ER), nuclear envelope, and unidentified vesicles, but none interacted with mitochondria (Sattarzadeh et al., 2011). Inspection of the members of the Arabidopsis myosin XI gene family revealed that except for XI-J, Arabidopsis myosin XI genes encode a 42 amino-acid region within the tail region that is homologous to the yeast myo2p region known to affect vacuole and mitochondrial inheritance (Figure 1). All of these regions in Arabidopsis exhibit the sequence PAL in this region; therefore we will refer to these 42 amino acids as the Arabidopsis myosin XI PAL sub-domains. We have previously expressed a YFP fusion to the PAL sub-domain of myosin XI-F and have shown that it localizes to plastids, stromules, peroxisomes, and Golgi in particular cells (Sattarzadeh et al., 2009). Here we report that YFP fusions of the PAL domain from 11 additional Arabidopsis myosin XIs localize to Golgi, mitochondria, nuclear envelope, the plasma membrane and/or to unidentified vesicles. While Golgi and mitochondrial mobility continued in cells transiently expressing the PAL sub-domains of either myosin XI-H, XI-I, and XI-K, simultaneous expression of all three PAL sub-domains resulted in cells with minimal or negligible movement of the two organelles.

## Results

### Arabidopsis myosin XI tail domain homologous to a yeast myosin V domain

In the yeast myosin ScMyo2p, there is a 11 amino acid section that has been shown by mutagenesis to be critical for vacuole inheritance (Catlett et al., 2000). Mutations in this region also affect mitochondrial inheritance (Fortsch et al., 2011). A 42 amino acid region encompassing the 11 amino acids is highly conserved between yeast and plants and among the 12 different plant myosin XIs (Figure 1B). Based on the ScMyo2p structure template (PDB code2F6HX), homology models of globular tails of all *A. thaliana* class XI myosins were built. The three- dimensional structures that were obtained indicate that the predicted general architecture of the PAL sub-domain in class XI myosins is very similar to that of ScMyo2p (Figure S1; Figure 1C). The models indicate that the 11 amino-acid sections homologous to the corresponding yeast sections are predicted to be exposed on the surface of the polypeptides when the 42-amino acid regions we expressed are modeled.

### Localization of YFP::at myosin XI PAL sub-domain fusions

Previously we have fused the PAL sub-domain from Arabidopsis myosin XI-F to YFP and have shown that the fusion protein labels plastids and stromules (Sattarzadeh et al., 2009). We have now made constructs with the remaining 11 PAL sub-domains from the Arabidopsis class XI myosins fused to YFP at the N-terminus. We report YFP fusion information for XI-C and XI-E together because the PAL domains of these two myosin XIs are identical. The localization of the YFP fusion proteins was examined by confocal laser scanning microscopy (CLSM) in leaf epidermal cells of *N. benthamiana* following agroinfiltration.

**Figure 2:**
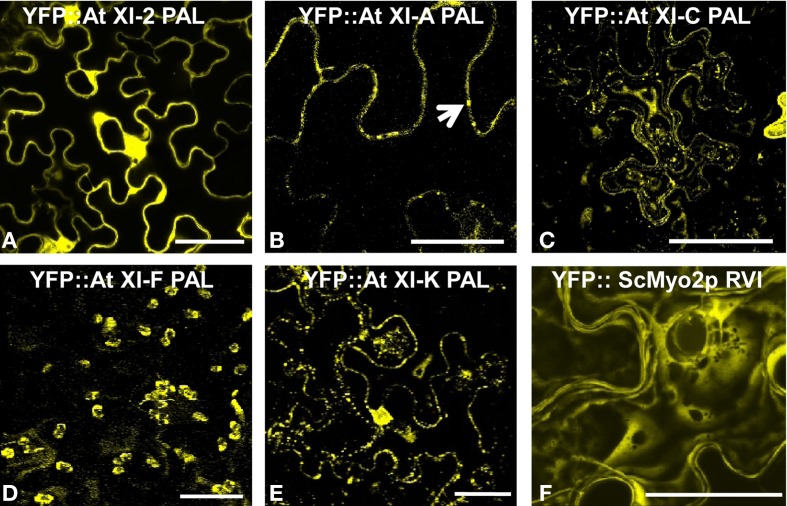
**Transient expression of YFP:: Myosin PAL sub-domains.** Agrobacterium-mediated transient expression of YFP fusions of PAL sub-domains in epidermal cells of *N. benthamiana* **(A)** At XI-2. **(B)** At XI-A. **(C)** At XI-C. **(D)** At XI-F. **(E)** XI-K. **(F)** ScMyo2p RVI (Required for Vacuole Inheritance). An arrow marks some of the vesicles observed with YFP:: At XI-A PAL. Scale bar = 50 μm.

Except for the YFP::At XI-2 PAL fusion, all tested fusions labeled vesicles in the same size range as the Golgi, endosomes, and mitochondria (Figures 2A–E). The YFP fusions with myosin XI-2 failed to localize to any vesicular structures and strongly labeled the plasma membrane (Figure 2A; Figure S2). The YFP fusion with the At XI-A PAL sub-domain was usually seen also in the cytoplasm; however, some unidentified vesicular structures were sometimes observed in a few cells (Figure 2B). YFP fusion with the homologous region in yeast (RVI: Required for Vacuole Inheritance) resulted in diffuse cytoplasmic labeling in plant cells, but some punctate loci could be discerned at the plasma membrane (Figure 2F).

**Figure 3:**
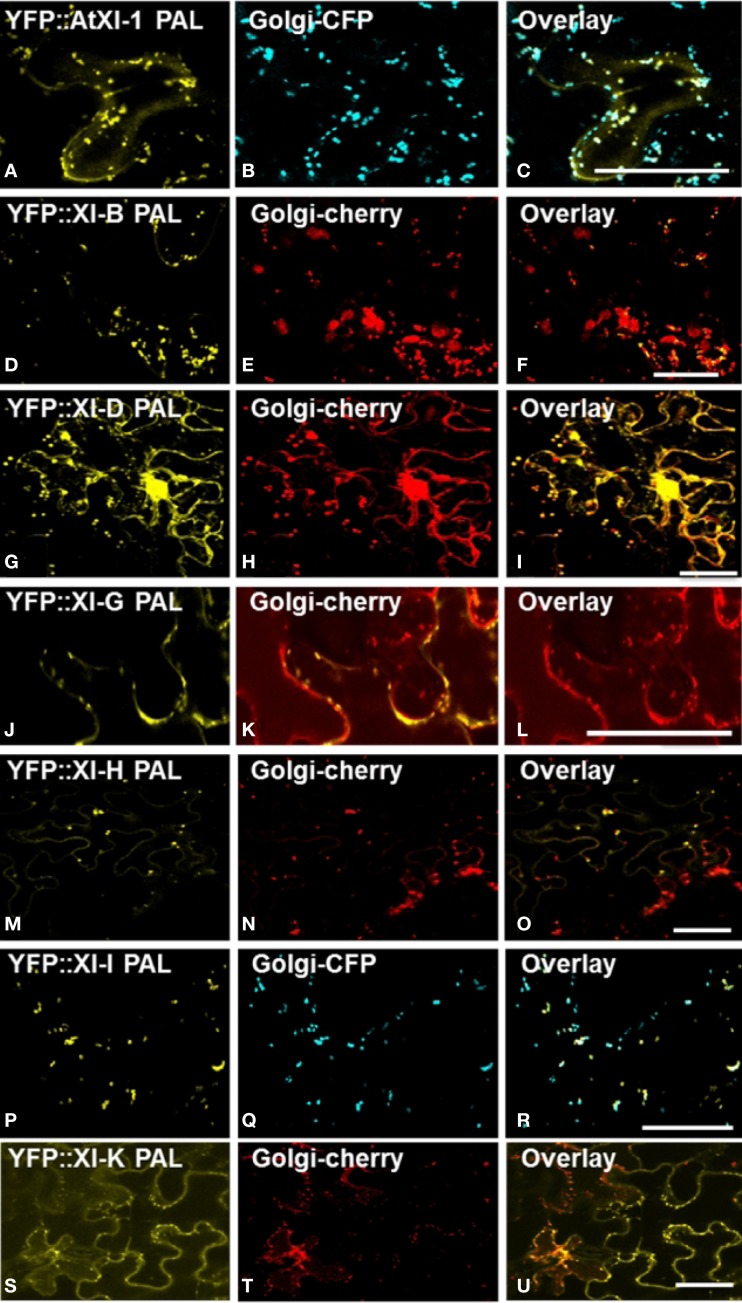
**Co-expression of YFP::Myosin XI PAL sub-domain with CFP and mCherry markers for Golgi in epidermal leaf cells of *N. benthamiana* (A) At XI-1. (D)** At XI-I B. **(G)** At XI-D. **(J)** At XI-G. **(M)** At XI-H. **(P)** At XI-I **(S)** At XI-K. **(B,E,H,K,N,Q,T)** Golgi marker. **(C,F,I,L,O,R,U**) Merged images of YFP (yellow) and mCherry (red) or CFP (blue). Scale bar = 50 μm

In order to identify the YFP-labeled organelles, we performed co-localization studies by co-expressing the myosin YFP-PAL fusions transiently with CFP or mCherry organelle markers in leaf epidermal cells of *N. benthamiana*. The YFP fusions with the PAL sub-domain of myosins XI-1, XI-D, XI-G, XI-H, XI-I and XI-K co-localized with the Golgi marker (Figure 3). Some but not all Golgi were labeled by the YFP::PAL fusion of myosin XI-B (Figures 3D–F). Vesicles labeled by YFP fusions with the PAL sub-domain of XI-C and XI-E did not coincide with the Golgi marker (data not shown).

**Figure 4:**
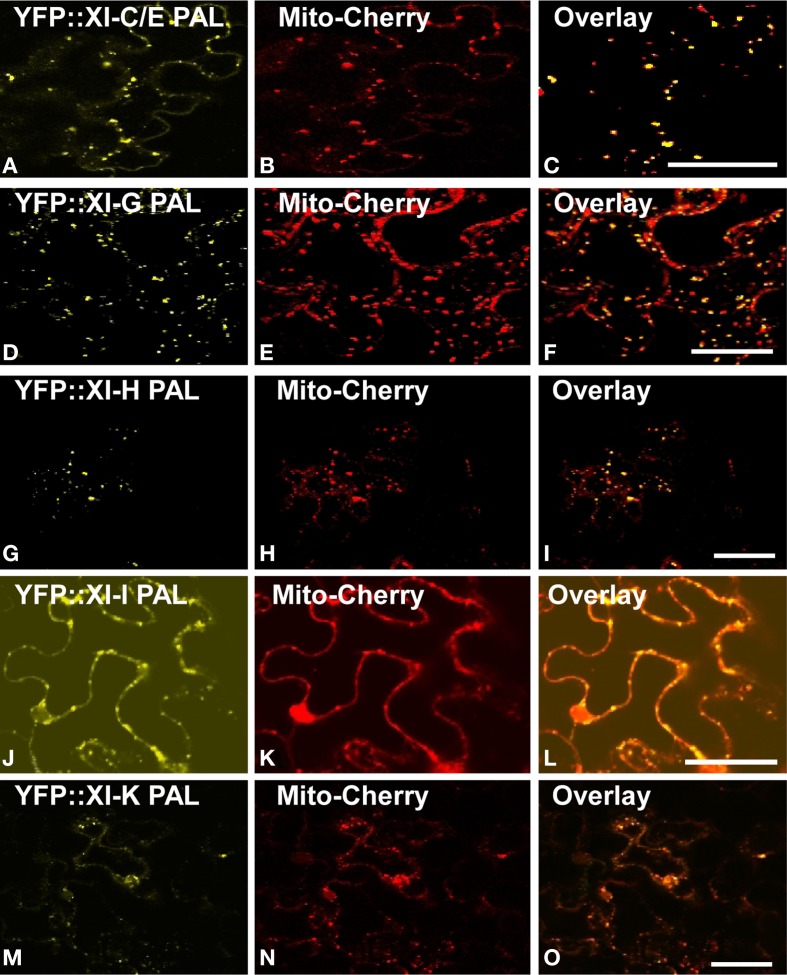
**Co-Expression of YFP::Myosin XI PAL sub-domain with mCherry markers for mitochondria in epidermal leaf cells of *N. benthamiana*. (A)** At XI-C. **(D)** At XI-I G. **(G)** At XI-H. **(J)** At XI-I **(M)** At XI-K. **(B,E,H,K,N)** mCherry mitochondrial marker. **(C,F,I,L,O)** Merged images of YFP (yellow) and mCherry (red). Scale bar = 50 μm.

**Figure 5:**
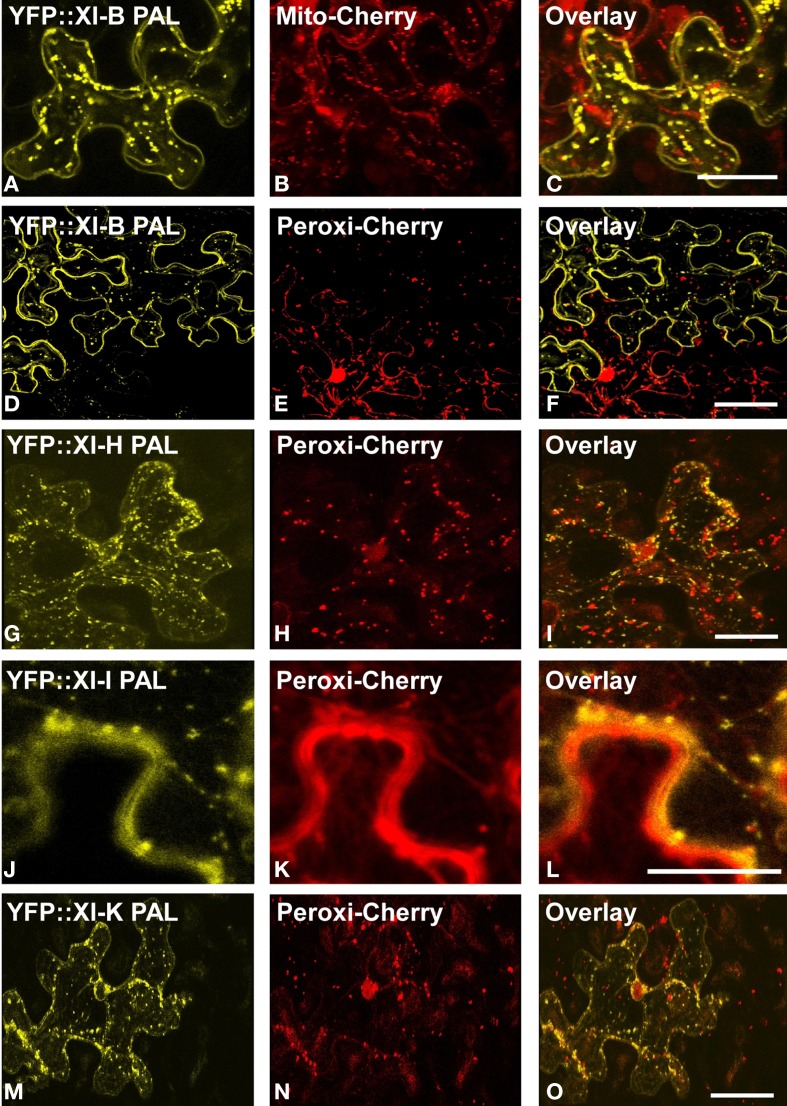
**Lack of co-localization with mitochondrial and peroxisome markers.** Co-expression of YFP::Myosin XI PAL sub-domains with mCherry markers for mitochondria and peroxisomes in epidermal leaf cells of *N. benthamiana*. **(A** and **D**) At XI-B. **(G)** At XI-H. **(J)** At XI-I **(M)** At XI-K. **(B)** mCherry mitochondrial marker. **(E,H,K,N)** mCherry peroxisomes. **(C,F,I,L,O)** Merged images of YFP (yellow) and mCherry (red). Scale bar = 50 μm

YFP fusions with the PAL sub-domain from myosins XI-C, XI-E, XI-G, XI-H, XI-I, and XI-K co-localized with a mitochondrial marker (Figure 4). YFP::PAL fusions with myosins XI-B (Figure 5) and XI-D (data not shown) did not coincide with the mitochondrial signal. When YFP::PAL fusions of myosin XI-B, XI-H, XI-I, and XI-K were expressed along with a peroxisomal marker, no co-localization was observed (Figure 5).

**Figure 6:**
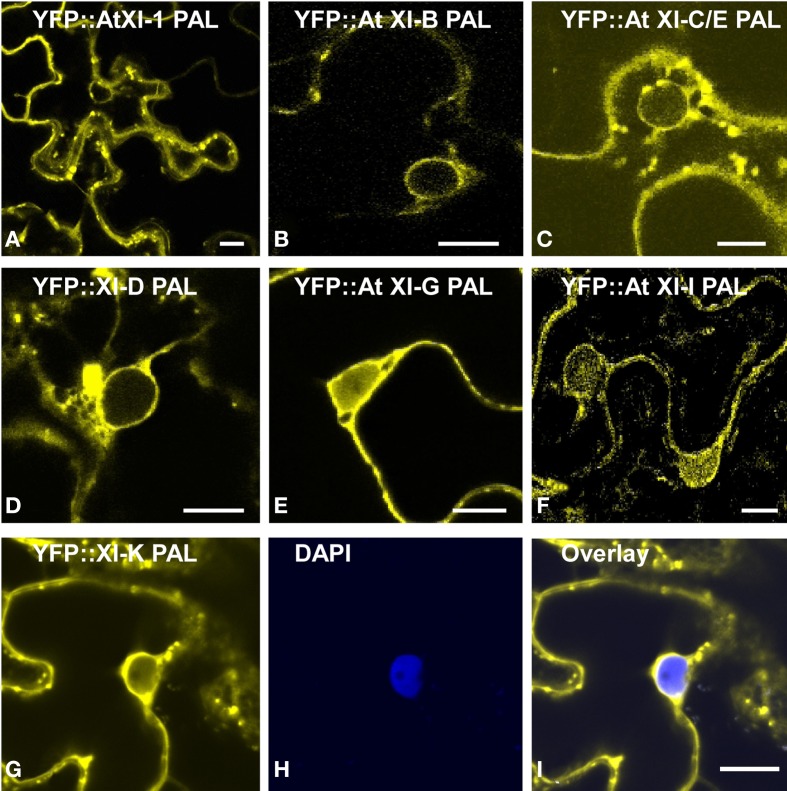
**Transient expression of YFP fusions of *A. thalian*a class XI myosin PAL sub-domains in epidermal leaf cells of *N. benthamiana*.** YFP fusions of At XI-1 PAL **(A)**, At XI-B PAL **(B)**, At XI-C/E PAL **(C)**, At XI-D PAL **(D)**, At XI-G **(E)**, At XI-I PAL **(F)** and At XI-K PAL **(G)** are shown in yellow. **(H)** DAPI staining of epidermal leaf cells of N. benthamiana expressing YFP:: At XI-K PAL. **(I)** Merged images of YFP (yellow) and DAPI staining (blue). Scale bar = 15 μm.

**Figure 7:**
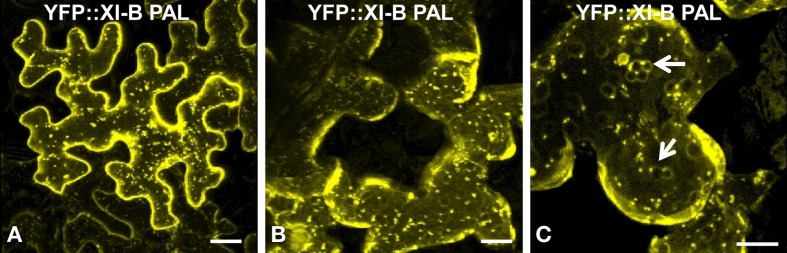
**Transient expression of YFP::At XI-B PAL in epidermal leaf cells of *N. benthamiana*. (A)** In most cells, this YFP pattern was observed. **(B and C**) Occasionally, structures characteristic of tonoplasts (Xu et al., 2006) were observed. Examples of the putative tonoplasts are indicated with arrows. Scale bar = 25 μm.

Eight of the YFP fusions to PAL sub-domain (AtXI-1,B,C,D,E,G,I and K) appear to label the nuclear envelope in epidermal cells (Figures 6A–I). Even though the homologous region in yeast interacts with vacuoles, we observed only one plant PAL sub-domain, from XI-B, that label structures that are characteristic of tonoplast membranes in appearance (Xu et al., 2006) (Figure 7). Most cells expressing XI-B exhibited fluorescent Golgi, vesicles, and nuclear envelopes (Figure 7). The positive and negative co-localizations are summarized in Table 1.

**Table 1:** **Summary of localization of PAL sub-domains of *A. thaliana* class XI myosins N-terminally fused to YFP and transiently expressed in leaves epidermal cells of *N. benthamiana***.

**Class XI**	**PAL sub-domain colocalization**	**PAL domain No colocalization**
At XI-1	Golgi	
	Nuclear envelope	
At XI-2	Plasma membrane	
At XI-A	Cytoplasm, Vesicles	
At XI-B	Golgi	*Golgi (some)*,	
	Nuclear envelope	*Mitochondria*,
	Vesicles	*Peroxisomes*
	Plasma membrane	
	Tonoplast	
At XI-C/E	Mitochondria	*Golgi*
	Nuclear envelope	
At XI-D	Golgi,	*Mitochondria*
	Nuclear envelope	
At XI-F	Plastids, Stromules	
	Nuclear envelope Golgi, Peroxisomes	
At XI-G	Mitochondria, Golgi	
	Nuclear envelope	
At XI-H	Golgi, Mitochondria	*Peroxisome*
At XI-I	Golgi, Mitochondria	*Peroxisome*
	Nuclear envelope	
At XI-K	Golgi, Mitochondria, Nuclear envelope	*Peroxisome*

*Organelles that were observed not to co-localize with a particular myosin are shown in italics*.

### Motility of the vesicles labeled with YFP::PAL sub-domain fusions

**Figure 8:**
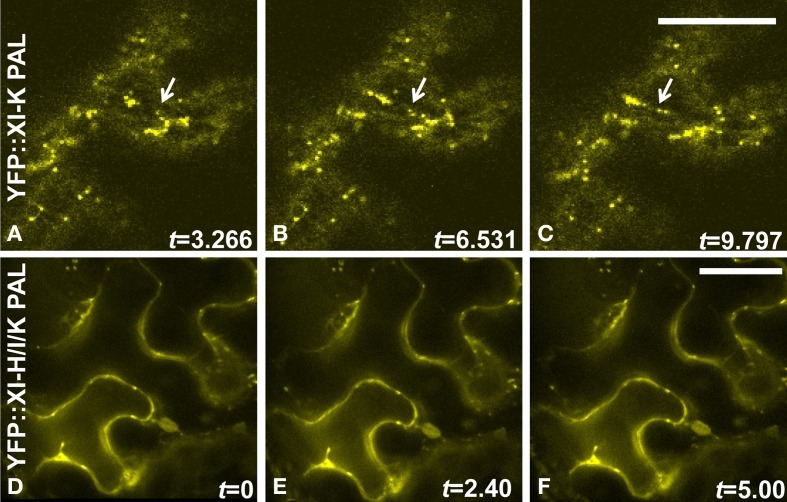
**Sequential images indicating movement YFP::At XI-K PAL sub-domain-labeled organelles but lack of movement when multiple domains are infiltrated simultaneously. (A–C)** *N. benthamiana* leaves were infiltrated with an Agrobacterium strain carrying a YFP::At XI-K PAL sub-domain. **(D–F)** Leaves were infiltrated simultaneously with three Agrobacterium strains (YFP::At XI-H, YFP::At XI-I, and YFP::At XI-K PAL sub-domains), each of which contained a transgene with one PAL sub-domain fused to YFP. Time-lapse series were obtained by confocal microscope. Only three frames are shown. Time (*t*) is indicated in seconds. Bar = 50 um. An arrow marks an organelle that is in different positions in **A–C**.

High levels of expression are often achieved following agroinfiltration. When a myosin tail domain lacking the motor function is expressed, a dominant negative effect on motility can sometimes be detected. However, when we examined the vesicles labeled by the 11 YFP::At PAL sub-domains, we found that rapid motility was retained, in contrast to our prior observation of stationary vesicles following agroinfiltration of YFP::At DIL domain fusions. Motility is documented in Figures 8A–C and Movies S1–S4 in Supplementary Material. In order to determine whether a dominant negative effect on movement might occur if multiple PAL sub-domains were expressed, we agroinfiltrated simultaneously four strains, containing YFP-PAL sub-domain fusions from myosins XI-H, XI-I, and XI-K, along with a strain carrying either a mCherry marker for Golgi or for mitochondria. About a third of the cells expressing YFP exhibited YFP-labeled vesicles that were stationery or poorly motile (Figures 8D–F). Agroinfiltration does not result in all cells in a leaf expressing the transgenes, so some cells may be expressing only one or two of the PAL sub-domain fusions. We show an example of two adjacent cells, one of which is expressing one or more YFP-labeled PAL sub-domains localized to nearly immobile vesicles, while an adjacent cell is expressing only the mCherry mitochondrial marker and therefore exhibits motile mitochondria (Movie S5). We provide an example of a cell expressing mCherry-Golgi and YFP:: PAL sub-domains located on immobile vesicles in Movie S6. When the mCherry Golgi marker is infiltrated by itself, the Golgi exhibit high motility (Movie S7). We also observed minimal motility of YFP-labeled vesicles in many cells when we co-infiltrated three strains containing YFP-PAL sub-domain fusions from myosins XI-H, XI-I, and XI-K, without simultaneous co-infiltration of a strain carrying an organelle marker (Movie S8). In Figure 8, we provide three panels (A–C) from a movie that illustrate movement of vesicles labeled with YFP:: XI-K PAL when it is expressed alone; however, in (Figures 8D–F), no movement of vesicles occurs following co-infiltration of XI-H, XI-I, and XI-K. We conclude that expression of multiple PAL sub-domains can impair movement of Golgi and mitochondria by a dominant negative effect.

## Discussion

**Figure 9:**
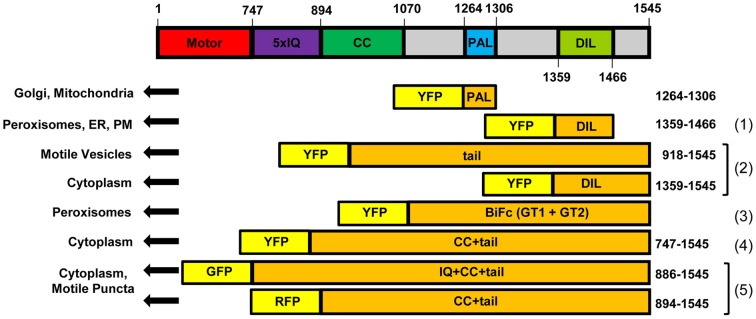
**Diagram of At XI-K constructs that have been expressed in Nicotiana.** Constructs diagramed are (1) from Sattarzadeh et al. (2011), (2) from Reisen and Hanson (2007), (3) from Li and Nebenfuhr (2007), (4) from Sparkes et al. (2008), and (5) from Avisar et al. (2009). CC: coiled-coil. IQ: IQ repeats. BiFC: construct for Bimolecular Fluorescence Complementation. PM: Plasma Membrane.

We have observed that 42-amino acid portions of 12 Arabidopsis myosin XI tail regions (the PAL sub-domain) are targeted to different subcellular organelles and membranes. Previously Li and Nebenfuhr (2007) expressed larger regions of the Arabidopsis XI-1 and XI-2 tails that encompass the PAL sub-domain and found they were targeted to peroxisomes, unidentified vesicles, or remained in the cytoplasm (Figure 9). We recently reported that the DIL domain of Arabidopsis myosin XIs, which is C-terminal to the PAL sub-domain and also encompassed by the constructs used by Li and Nebenfuhr (2007), targets YFP to several organelles and membranes (Sattarzadeh et al., 2011). PAL and DIL regions from particular myosin XIs can target YFP to Golgi, nuclear envelope, and/or plasma membrane. However, no DIL domains resulted in localization of YFP to mitochondria or tonoplast. Conversely, none of the PAL sub-domain fusions resulted in labeling of ER nor peroxisomes, while 8 DIL domains targeted YFP to peroxisomes and two labeled ER (Sattarzadeh et al., 2011). Thus there are some general differences in the localization information provided by the two different tail regions.

Although the PAL sub-domains among the *A. thaliana* class XI myosins are quite similar, there are some sequence differences in this 42 amino acid region of individual tail domains that could explain the different subcellular localizations (Table 2). While the PAL sub- domains of XI-C and XI-E are completely identical, other myosin XI PAL regions are less similar; nevertheless, it is not possible to consult the table of identity to predict which PAL sub-domains are likely to label one organelle vs. another. For example, of the PAL sub-domains that label mitochondria, XI-C/E PAL is 71, 80, 85, and 95% identical, respectively, to XI-G, XI-H, XI-I, and XI-K PAL. However, XI-C/E PAL is 85, 83, and 85%, identical, respectively to XI-1, XI-B, and XI-F, which do not label mitochondria but do label Golgi (Table 2). Unidentified aspects of the protein tertiary structure may be important for selective labeling.

**Table 2:** **Pairwise comparison of the amino acid sequences of the PAL sub-domain from *A. thaliana* class XI myosins and vacuole binding site in the yeast class V myosin Myo2p**.

	**Myo2P**	**AtMYA1**	**AtMYA2**	**AtXI-A**	**AtXI-B**	**AtXI-C**	**AtXI-D**	**AtXI-E**	**AtXI-F**	**AtXI-G**	**AtXI-H**	**AtXI-I**	**AtXI-K**
Myo2P	42	16%	11%	9%	16%	16%	14%	16%	16%	19%	14%	19%	16%
AtMYA1	7	42	69%	66%	73%	85%	59%	85%	76%	61%	69%	78%	90%
AtMYA2	5	29	42	71%	85%	80%	59%	80%	73%	69%	83%	71%	76%
AtXI-A	4	28	30	42%	66%	71%	78%	71%	59%	59%	66%	71%	66%
AtXI-B	7	31	36	28	42	83%	57%	83%	73%	69%	80%	76%	78%
AtXI-C	7	36	34	30	35	42	61%	100%	83%	71%	80%	85%	95%
AtXI-D	6	25	25	33	24	26	42	61%	50%	59%	59%	64%	59%
AtXI-E	7	36	34	30	35	42	26	42	83%	71%	80%	85%	95%
AtXI-F	7	32	31	25	31	35	21	35	42	64%	69%	69%	78%
AtXI-G	8	26	29	25	29	30	25	30	27	42	76%	64%	66%
AtXI-H	6	29	35	28	34	34	25	34	29	32	42	71%	76%
AtXI-I	8	33	30	30	32	36	27	36	29	27	30	42	83%
AtXI-K	7	38	32	28	33	40	25	40	33	28	32	35	42

*The identity is shown in percent and the number of identical amino acids are listed*.

The PAL sub-domain of the myosin XIs was selected for study because it is homologous to a sub-domain in yeast myo2p required for vacuole inheritance (Catlett et al., 2000). Recently, (Fortsch et al., 2011) have shown that the same yeast myo2p region is important for mitochondrial inheritance. Furthermore, Li and Nebenfuhr (2007) demonstrated that the structure of myosin XI-1 could be modeled with structural data available for yeast myo2p. Ancestral myosin XIs may have duplicated and diverged in the tail domains to interact with a variety of subcellular structures, including mitochondria, Golgi, and peroxisomes. The yeast PAL sub-domain did not label plant mitochondria or tonoplast (Figure 2F), though the yeast DIL domain was able to label plant peroxisomes (Sattarzadeh et al., 2011).

High-level expression of defective motor proteins in various organisms have often been observed to create a dominant-negative effect on organelle movement, possibly because saturation of receptors with the motorless protein prevents intact motors from binding. When we transiently expressed YFP::DIL fusions of 12 Arabidopsis myosin XI proteins, we found that expression of all but one of the fusions resulted in loss of motility of YFP-labeled vesicles and organelles (Sattarzadeh et al., 2011). Expression of various portions of fluorescent protein fusions to myosin XI tails have sometimes resulted in detectable effects on motility even though the affected organelles were not visibly fluorescently labeled. For example, Avisar et al. (2009) did not document localization of GFP::myosin XI tail regions to Golgi or mitochondria, but did detect inhibition of movement of both of the organelles when fusions to XI-1, XI-2, XI-C, XI-E, XI-I, and XI-K were expressed in *N. benthamiana*. Vick and Nebenfuhr (2012) have pointed out that expression of myosin tail regions might result in non-specific inhibition of motility as well as receptor-specific inhibition. We detected no Golgi or mitochondrial labeling with either the PAL or DIL regions of XI-2, but YFP::PAL of XI-C and E labeled mitochondria, YFP: DIL of XI-E labeled peroxisomes and YFP::DIL of XI-1 labeled Golgi (Figure 3 and Sattarzadeh et al., 2011).

We have expressed Arabidopsis PAL domains in a heterologous plant system, namely *N. benthamiana*. The PAL domains in this species are highly similar to those in Arabidopsis (Figure 1; Figure S3). In fact, there are *N. benthamiana* PAL domains that are identical to those in Arabidopsis XI-K and XI-C/E (Figure S3). Thus proteins present in *N. benthamiana* that bind to PAL domains of endogenous myosin XIs are likely to be able to interact strongly with expressed Arabidopsis PAL domains. However, divergence between some of the Arabidopsis and Nicotiana PAL domains may result in weaker or absent interaction with Nicotiana endogenous proteins, potentially explaining why expression of a single Arabidopsis PAL domain is not sufficient for a strong dominant-negative effect. Furthermore, we may not have detected all the subcellular structures that interact with Arabidopsis PAL domains in Arabidopsis, if they are so divergent that they cannot interact with the myosin receptor proteins in Nicotiana.

Sparkes et al. (2008) were able to observe fluorescent puncta when YFP fusions to tail regions of Arabidopsis XI-E and XI-K were expressed in *N. tabacum* epidermal cells. Although not all peroxisomes, mitochondria, and Golgi were labeled by the YFP fusions to XI-E and XI-K, inhibition of their movement was detected in the cells (Sparkes et al., 2008). We observed that the YFP::PAL sub-domain fusions of XI-I and XI-K labeled mitochondria and Golgi in *N. benthamiana*, but YFP::PAL XI-H also labeled both organelles. Our YFP::PAL XI-B, XI-F, and XI-H fusions labeled Golgi. This is consistent with the observations of Avisar et al. (2009), who observed inhibition of Golgi movement by fusions with XI-B, XI-F, and XI-H; however, the inhibition occurred only in *N. tabacum* and not in *N. benthamiana*. Thus if a PAL sub-domain labels a particular motile organelle, the larger constructs expressed by others have often resulted in effects on motility of that organelle. But the correlation is not perfect, as we did not detect labeling of all organelles whose movement was affected by expression of a particular myosin tail. For example, although Sparkes et al. (2008) observed inhibition of Golgi by expression of YFP::XI-E tail fusions, neither the PAL nor DIL regions of XI-E labeled Golgi, even though the XI-E PAL sub-domains in *A. thaliana* and *N. benthamiana* are identical. Possibly other sequences we have not analyzed are able to interact with Golgi and mitochondria; however, abnormal conformation of myosin fragments may also prevent interactions that would normally occur with the intact myosin.

Single myosin XI insertional mutants do not exhibit major phenotypes, though XI-2 and XI-K knockouts are affected in root hair elongation and in Golgi, mitochondrial, and peroxisome motility (Peremyslov et al., 2008). While we have not been able to observe labeling of these three organelles with YFP fusions with XI-2 PAL sub-domains, all three organelles are labeled by either the PAL sub-domain (mitochondria, Golgi) or the DIL domain (peroxisomes) of XI-K fused to YFP. In plants containing single knockouts of XI-1, XI-2, XI-C, XI-E, and XI-I, Avisar et al. (2012) detected minor effects on Golgi movement, while an XI-K knockout greatly inhibited Golgi motility. Of these myosins, we found that YFP::PAL sub-domain fusions of XI-1, XI-I, and XI-K and the YFP::DIL fusion of XI-C labeled Golgi (Sattarzadeh et al., 2011).

Of the Arabidopsis myosin XIs, XI-1, XI-2, XI-H, XI-I, and XI-K are the most highly and ubiquitously expressed (Peremyslov et al., 2011; Sparkes, 2011). Much attention has been focused on myosin XI-K. Avisar et al. (2008b) observed that transient silencing or expression of dominant negative constructs of myosin XI-K resulted in impaired movement of Golgi, peroxisomes, and mitochondria. More recent experiments with the XI-K tail region of *N. benthamiana* demonstrated that the particular myosin fragment that is expressed greatly influences how much vesicle motility is affected (Avisar et al., 2012). When Avisar et al. (2012) expressed two globular tail regions that encompass the corresponding Arabidopsis XI-K PAL and DIL domains that we have utilized, they observed inhibition of Golgi movement, but a third construct with a C-terminal extension, and two single amino-acid mutations in the DIL domain resulted in retention of Golgi motility (Avisar et al., 2012). When the entire XI-K sequence was fused to YFP, a small fraction of the Golgi, some regions of the ER, and many endomembrane vesicles were labeled (Peremyslov et al., 2012). The importance of myosin XI-K is also indicated in our YFP fusion experiments, as XI-K PAL or DIL regions fused to YFP resulted in labeling of many locations: Golgi, mitochondria, peroxisomes, nuclear envelope, plasma membrane, and ER (Figures 3, 4; Sattarzadeh et al., 2011).

Even though expression of 12 different Arabidopsis YFP::PAL sub-domain fusions resulted in readily detectable fluorescent labeling of various subcellular structures, the labeled Golgi and mitochondria move rapidly (Movies S1–S4). We have identified 6 different myosin XIs whose PAL sub-domains can interact with mitochondria, and 7 myosin XIs whose PAL sub-domains can interact with Golgi. When certain tail regions of 6 myosin XIs were labeled in transient assays, we previously observed that the motility of vesicles was retained (Reisen and Hanson, 2007). In contrast, when we expressed YFP::DIL fusions in *N. benthamiana*, movement of Golgi and peroxisomes ceased (Sattarzadeh et al., 2011). We cannot rule out the possibility that some of the targeting of YFP::PAL sub-domains to organelles may be due to chance function of the 42 amino-acid sequence as a transit peptide or secretory signal, as C-terminal fusions can sometimes unexpectedly result in labeling of peroxisomes and other vesicles (Cutler et al., 2000). However, targeting programs do not predict any of the PAL sub-domains to be effective transit or signal sequences (Table S1 in Supplementary Material), though we recognize that such predictions are not perfect. The organelles labeled by the myosin XI PAL sub-domains are largely consistent with a role for the individual myosin XIs in organelle motility, according to the results of mutant, silencing, and dominant negative analysis. Possibly the YFP::PAL sub- domains do not accumulate to the same levels as the YFP::DIL fusion, thus not saturating receptors, allowing a sufficient number of intact motors to attach and transport the organelles. Alternatively, there may be differences in the Golgi and mitochondrial receptors that interact with the multiple PAL sub-domains, so that even if one receptor is saturated with a defective myosin, other receptors can interact with other myosins. Different myosin XIs may vary in affinity for particular cargoes, especially in a heterologous system. Less than 7% of the Golgi were labeled with a full-length myosin XI-K::YFP fusion (Peremyslov et al., 2012). We did not observe a dominant negative effect on Golgi and mitochondrial motility until we expressed three PAL sub-domains (XI-H, XI-I, XI-K) simultaneously (Movies S5, S6, S8). Further inquiry into the role of the PAL sub-domains is needed in order to determine whether motility of Golgi and mitochondria is affected as a result of saturation of receptors on the cargo or because of unknown non-specific effects on myosin function.

## Materials and methods

### Generation of YFP::myosin constructs, plant growth and agroinfiltration

Generation of the YFP::myosin constructs was performed as described (Sattarzadeh et al., 2009) using standard TOPO and Gateway cloning system (*Invitrogen*, Carlsbad, CA). The primers used are listed in Table S2 in Supplementary Material. Plant growth and agroinfiltration was done as described (Sattarzadeh et al., 2011). When multiple strains were infiltrated, the same total concentration of bacteria was used as for single infiltration.

### Fluorescent labeling of organelles and confocal laser scanning microscopy

For labeling of mitochondria, peroxisomes, Golgi stacks and plasma membrane with mCherry fluorescent protein, binary vectors, CD3-991, CD3-984, CD3-967, and CD3-1008 (Nelson et al., 2007) were used, respectively. According to Nelson et al. (2007), the mitochondrial marker was made by fusing the first 29 amino acids from the N-terminus of yeast COXIV (Köhler et al., 1997) to mCherry, the peroxisomal targeting signal Ser-Lys-Leu (Reumann, 2004) to mCherry, and the Golgi marker resulted from fusion of the first 49 amino acids of soybean α-1,2-mannosidase I (Saint-Jore-Dupas et al., 2006) to mCherry. The Golgi CFP marker was made by Sattarzadeh et al. (2011) by fusing the first 52 N-terminal amino acids of a rat 2, 6-sialyl transferase (Boevink et al., 1998). For DNA staining by 4′,6-diamidino-2-phenylindole (DAPI), leaves were immersed in a solution of 2.5% mannitol/0.01%silwet/300 nM DAPI.

CSLM was performed on a Leica microscope equipped with a TCS-SP2 confocal scanning head (Leica Microsystems, Heidelberg, Germany) as described (Sattarzadeh et al., 2009). Movies S5–S7 were captured by using an Andor XD revolution spinning disc confocal microscope. Infiltrations of each YFP::PAL fusion were performed at 3 separate times and approximately 100 cells in total were observed for each YFP::PAL fusion. The images shown represent the appearance of the majority of the labeling patterns for each fusion. We note in the text if an image is provided of labeling pattern that was only rarely observed.

### Bioinformatic analysis and structural homology model analysis

GeneDoc was used for generation of alignments (Nicholas and Nicholas, 1997). Percent identity and divergence between Arabidopsis and *N. benthamiana* myosin XI PAL domains was generated with the LASERGENE MEGALIGN program (DNAstar, Madison, WI). Structure homology models for the tail domain of class XI mysoins were built by SWISSMODEL (Arnold et al., 2006) based on the Myo2p structure template (2f6hX). PyMol (Delano Scientific) was used for drawing the structural homology models.

### Conflict of interest statement

The authors declare that the research was conducted in the absence of any commercial or financial relationships that could be construed as a potential conflict of interest.

## Supplementary material

The Supplementary Material for this article can be found online at:

http://www.frontiersin.org/Journal/10.3389/fpls.2013.00407/abstract

Figure 1 **Homology structural modeling of myosin XI globular tail based on similarity to the myosin V ScMyo2p**. Predicted three-dimensional structures of the globular tail of *A. thaliana* class XI myosins are illustrated. **(A)** XI-1. **(B**) XI-2. **(C)** XI-A. **(D)** XI-B. **(E)** XI-C. (**F**) XI-D. **(G)** XI-E. **(H)** XI-F. **(I)** XI-G. **(J)** XI-H. **(K)** XI-I. **(L)** XI-K. Surface residues are shown in orange. The 11 amino acids critical for the vacuole binding site in ScMyo2p tail (Catlett et al., 2000) and the corresponding amino acids in PAL sub-domains are shown in cyan.

Figure 2 **Co-expression of YFP::Myosin XI-2 PAL domain with mCherry markers for the plasma membrane in epidermal leaf cells of *N. benthamiana* (A) At XI-2 (B) Plasma membrane marker. (C)** Merged images of YFP (yellow) and mCherry (red). Scale bar = 10 μm.

Figure 3 **Pairwise comparison of the amino acid sequences identity and divergence between Arabidopsis and *N. benthamiana* myosin XI PAL domains**. Table of identity and divergence was generated with the LASERGENE MEGALIGN program(DNAstar, Madison, WI).

Movies S1–S4 **Movement of YFP::At XI-PAL sub-domain-labeled organelles**. *N. benthamiana* leaves were infiltrated with an Agrobacterium strain carrying a YFP::AtXI-PAL sub-domain **(S1)** XI-1 **(S2)** XI-C/E **(S3)** XI-D and **(S4)** XI-K.

Movie S5 **Co-expression of YFP::At XI-H, YFP::At XI-I and YFP::AT XI-K PAL sub-domains with mCherry markers) for mitochondria in epidermal leaf cells of *N. benthamiana* leaves**. Leaves were infiltrated simultaneously with four Agrobacterium strains, each of which contained either contained a transgene with one PAL sub-domain fused to YFP (yellow) or a mCherry protein (red) targeted to mitochondria. Time-lapse series (25 frames, 0.2 s intervals) were obtained with an Andor spinning disc confocal microscope using a 60X objective. To the right of the yellow-labeled cell is a cell with motile mCherry-labeled mitochondria that is not exhibiting expression of the YFP-labeled PAL sub-domains.

Movie S6 **Co-expression of YFP::At XI-H, YFP::At XI-I and YFP::AT XI-K PAL sub-domains with mCherry markers (red) for Golgi in epidermal leaf cells of *N. benthamiana* leaves**. Leaves were infiltrated simultaneously with four Agrobacterium strains, each of which contained either contained a transgene with one PAL sub-domain fused to YFP (yellow) or a mCherry protein (red) targeted to mitochondria. Time-lapse series (25 frames, 0.2 s intervals) were obtained with an Andor spinning disc confocal microscope using a 60X objective.

Movie S7 **Movement of mCherry-labeled Golgi bodies in epidermal cells of *N. benthamiana* leaves**. Time-lapse series (20 frames, 3 s intervals) were obtained by the Leica CSLM. The movie is shown at 5 frames per second.

Movie S8 **Co-expression of YFP::At XI-H, YFP::At XI-I and YFP::AT XI-K PAL sub-domains in epidermal leaf cells of *N. benthamiana* leaves**. Leaves were infiltrated simultaneously with three Agrobacterium strains, each of which contained a transgene with one PAL sub-domain fused to YFP. Time-lapse series (25 frames, 0.2 s intervals) were obtained with an Andor spinning disc confocal microscope using a 60X objective.

Table S1 **Predicted subcellular localization of *A. thaliana* class XI myosin PAL sub-domains**. The prediction was generated using Target P (Brunak et al., 2007) based on the predicted presence of any of the N-terminal presequences: chloroplast transit peptide (cTP), mitochondrial targeting peptide (mTP) or secretory pathway signal peptide (SP).

Table S2 **List of primers used in this study**.
