# Supplementary figures and images for: Arabidopsis myosin XI sub-domains homologous to the yeast myo2p organelle inheritance sub-domain target subcellular structures in plant cells
Source: Front Plant Sci. 2013 Oct 22;4:407. doi: 10.3389/fpls.2013.00407 (PMC3807578; doi:10.3389/fpls.2013.00407)

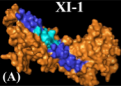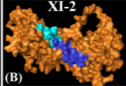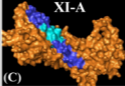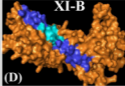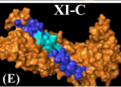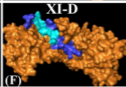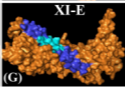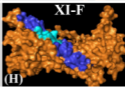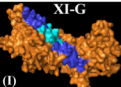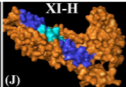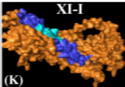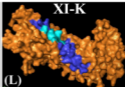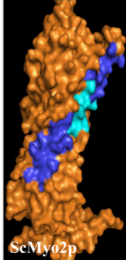

Supplement: Figure 1 — Homology structural modeling of myosin XI globular tail based on similarity to the myosin V ScMyo2p. Predicted three-dimensional structures of the globular tail of A. thaliana class XI myosins are illustrated. (A) XI-1. (B) XI-2. (C) XI-A. (D) XI-B. (E) XI-C. (F) XI-D. (G) XI-E. (H) XI-F. (I) XI-G. (J) XI-H. (K) XI-I. (L) XI-K. Surface residues are shown in orange. The 11 amino acids critical for the vacuole binding site in ScMyo2p tail (Catlett et al., 2000) and the corresponding amino acids in PAL sub-domains are shown in cyan. [file Presentation1.PDF]

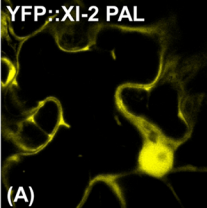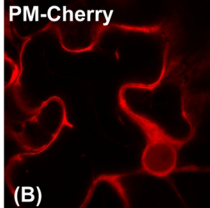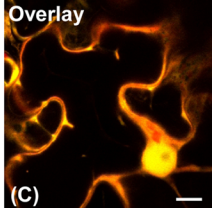

Supplement: Figure 2 — Co-expression of YFP::Myosin XI-2 PAL domain with mCherry markers for the plasma membrane in epidermal leaf cells of N. benthamiana (A) At XI-2 (B) Plasma membrane marker. (C) Merged images of YFP (yellow) and mCherry (red). Scale bar = 10 μm. [file Presentation2.PDF]
