# Supplementary material for: Arabidopsis myosin XI sub-domains homologous to the yeast myo2p organelle inheritance sub-domain target subcellular structures in plant cells
Source: Front Plant Sci. 2013 Oct 22;4:407. doi: 10.3389/fpls.2013.00407 (PMC3807578; doi:10.3389/fpls.2013.00407)
Supplement: Figure 3 — Pairwise comparison of the amino acid sequences identity and divergence between Arabidopsis and N. benthamiana myosin XI PAL domains. Table of identity and divergence was generated with the LASERGENE MEGALIGN program(DNAstar, Madison, WI). [file Presentation3.PDF]

Percent Identity

Divergence

	1	2	3	4	5	6	7	8	9	10	11	12	13	14	15	16	17	18	19	20	21	22	23	24		
1		69.0	66.7	73.8	85.7	59.5	85.7	76.2	61.9	69.0	78.6	90.5	90.5	90.5	85.7	85.7	85.7	85.7	78.6	76.2	76.2	73.8	73.8	76.2	1	AtXI-1
2	39.9		71.4	85.7	81.0	59.5	81.0	73.8	69.0	83.3	71.4	76.2	76.2	76.2	81.0	81.0	81.0	81.0	71.4	73.8	81.0	64.3	64.3	76.2	2	AtXI-2
3	43.9	36.0		66.7	71.4	78.6	71.4	59.5	59.5	66.7	71.4	66.7	66.7	66.7	71.4	71.4	71.4	71.4	64.3	66.7	76.2	61.9	61.9	76.2	3	AtXI-A
4	32.2	15.9	43.9		83.3	57.1	83.3	73.8	69.0	81.0	76.2	78.6	78.6	78.6	83.3	83.3	83.3	83.3	73.8	76.2	83.3	69.0	69.0	81.0	4	AtXI-B
5	15.9	22.0	36.0	18.9		61.9	100.0	83.3	71.4	81.0	85.7	95.2	95.2	95.2	100.0	100.0	97.6	97.6	85.7	88.1	88.1	81.0	81.0	83.3	5	AtXI-C
6	57.5	57.5	25.3	62.6	52.8		61.9	50.0	59.5	59.5	64.3	59.5	59.5	59.5	61.9	61.9	61.9	61.9	57.1	57.1	66.7	54.8	54.8	66.7	6	AtXI-D
7	15.9	22.0	36.0	18.9	0.0	52.8		83.3	71.4	81.0	85.7	95.2	95.2	95.2	100.0	100.0	97.6	97.6	85.7	88.1	88.1	81.0	81.0	83.3	7	AtXI-E
8	28.7	32.2	57.5	32.2	18.9	79.9	18.9		64.3	69.0	69.0	78.6	78.6	78.6	83.3	83.3	83.3	83.3	69.0	71.4	76.2	78.6	78.6	71.4	8	AtXI-F
9	52.8	39.9	57.5	39.9	36.0	57.5	36.0	48.2		76.2	64.3	66.7	66.7	66.7	71.4	71.4	71.4	71.4	61.9	64.3	76.2	61.9	61.9	71.4	9	AtXI-G
10	39.9	18.9	43.9	22.0	22.0	57.5	22.0	39.9	28.7		71.4	76.2	76.2	76.2	81.0	81.0	81.0	81.0	71.4	73.8	83.3	66.7	66.7	78.6	10	AtXI-H
11	25.3	36.0	36.0	28.7	15.9	48.2	15.9	39.9	48.2	36.0		83.3	83.3	83.3	85.7	85.7	83.3	83.3	85.7	90.5	81.0	78.6	78.6	81.0	11	AtXI-I
12	10.2	28.7	43.9	25.3	4.9	57.5	4.9	25.3	43.9	28.7	18.9		100.0	100.0	95.2	95.2	92.9	92.9	88.1	85.7	83.3	78.6	78.6	78.6	12	AtXI-K
13	10.2	28.7	43.9	25.3	4.9	57.5	4.9	25.3	43.9	28.7	18.9	0.0		100.0	95.2	95.2	92.9	92.9	88.1	85.7	83.3	78.6	78.6	78.6	13	Nb-Scf19118
14	10.2	28.7	43.9	25.3	4.9	57.5	4.9	25.3	43.9	28.7	18.9	0.0	0.0		95.2	95.2	92.9	92.9	88.1	85.7	83.3	78.6	78.6	78.6	14	Nb-Scf4550
15	15.9	22.0	36.0	18.9	0.0	52.8	0.0	18.9	36.0	22.0	15.9	4.9	4.9	4.9		100.0	97.6	97.6	85.7	88.1	88.1	81.0	81.0	83.3	15	Nb-Scf28722
16	15.9	22.0	36.0	18.9	0.0	52.8	0.0	18.9	36.0	22.0	15.9	4.9	4.9	4.9	0.0		97.6	97.6	85.7	88.1	88.1	81.0	81.0	83.3	16	Nb-Scf23426
17	15.9	22.0	36.0	18.9	2.4	52.8	2.4	18.9	36.0	22.0	18.9	7.5	7.5	7.5	2.4	2.4		100.0	83.3	85.7	88.1	81.0	81.0	83.3	17	Nb-Scf27533
18	15.9	22.0	36.0	18.9	2.4	52.8	2.4	18.9	36.0	22.0	18.9	7.5	7.5	7.5	2.4	2.4	0.0		83.3	85.7	88.1	81.0	81.0	83.3	18	Nb-Scf19220
19	25.3	36.0	48.2	32.2	15.9	62.6	15.9	39.9	52.8	36.0	15.9	13.0	13.0	13.0	15.9	15.9	18.9	18.9		95.2	76.2	71.4	71.4	71.4	19	Nb-Scf18993
20	28.7	32.2	43.9	28.7	13.0	62.6	13.0	36.0	48.2	32.2	10.2	15.9	15.9	15.9	13.0	13.0	15.9	15.9	4.9		78.6	76.2	76.2	73.8	20	Nb-Scf4030
21	28.7	22.0	28.7	18.9	13.0	43.9	13.0	28.7	28.7	18.9	22.0	18.9	18.9	18.9	13.0	13.0	13.0	13.0	28.7	25.3		71.4	71.4	95.2	21	Nb-Scf9118
22	32.2	48.2	52.8	39.9	22.0	68.0	22.0	25.3	52.8	43.9	25.3	25.3	25.3	25.3	22.0	22.0	22.0	22.0	36.0	28.7	36.0		100.0	69.0	22	Nb-Scf470
23	32.2	48.2	52.8	39.9	22.0	68.0	22.0	25.3	52.8	43.9	25.3	25.3	25.3	25.3	22.0	22.0	22.0	22.0	36.0	28.7	36.0	0.0		69.0	23	Nb-Scf40499
24	28.7	28.7	28.7	22.0	18.9	43.9	18.9	36.0	36.0	25.3	22.0	25.3	25.3	25.3	18.9	18.9	18.9	18.9	36.0	32.2	4.9	39.9	39.9		24	Nb-Scf30359
	1	2	3	4	5	6	7	8	9	10	11	12	13	14	15	16	17	18	19	20	21	22	23	24		
