# Supplementary material for: Arabidopsis myosin XI sub-domains homologous to the yeast myo2p organelle inheritance sub-domain target subcellular structures in plant cells
Source: Front Plant Sci. 2013 Oct 22;4:407. doi: 10.3389/fpls.2013.00407 (PMC3807578; doi:10.3389/fpls.2013.00407)
Supplement: Table S1 — Predicted subcellular localization of A. thaliana class XI myosin PAL sub-domains. The prediction was generated using Target P (Brunak et al., 2007) based on the predicted presence of any of the N-terminal presequences: chloroplast transit peptide (cTP), mitochondrial targeting peptide (mTP) or secretory pathway signal peptide (SP). [file DataSheet1.DOCX]

**TABLE S1. Predicted subcellular localization of *A.thaliana* class XI myosin PAL sub-domains.** The prediction was generated using Target P ([Brunak et al., 2007](#_ENREF_4)) based on the predicted presence of any of the N-terminal presequences: chloroplast transit peptide (cTP), mitochondrial targeting peptide (mTP) or secretory pathway signal peptide (SP).

|  | **cTP** | **mTP** | **SP** | **other** |
| --- | --- | --- | --- | --- |
| **Mya1** | **0.05** | **0.242** | **0.304** | **0.547** |
| **Mya2** | **0.037** | **0.324** | **0.319** | **0.397** |
| **XI-A** | **0.036** | **0.403** | **0.171** | **0.598** |
| **XI-B** | **0.03** | **0.224** | **0.44** | **0.447** |
| **XI-C** | **0.047** | **0.233** | **0.275** | **0.621** |
| **XI-D** | **0.029** | **0.249** | **0.337** | **0.657** |
| **XI-E** | **0.047** | **0.233** | **0.275** | **0.621** |
| **XI-F** | **0.059** | **0.235** | **0.227** | **0.559** |
| **XI-G** | **0.056** | **0.129** | **0.268** | **0.855** |
| **XI-H** | **0.044** | **0.179** | **0.423** | **0.531** |
| **XI-I** | **0.037** | **0.287** | **0.259** | **0.626** |
| **XI-K** | **0.038** | **0.221** | **0.39** | **0.528** |
