# Supplementary material for: Arabidopsis myosin XI sub-domains homologous to the yeast myo2p organelle inheritance sub-domain target subcellular structures in plant cells
Source: Front Plant Sci. 2013 Oct 22;4:407. doi: 10.3389/fpls.2013.00407 (PMC3807578; doi:10.3389/fpls.2013.00407)
Supplement: Table S2 — List of primers used in this study. [file DataSheet2.DOCX]

**TABLE S2.** List of primers used in this study.

| **Original name** | **Sequence** |
| --- | --- |
| At XI-1 PAL-F | 5´ (GWF) TCGTTGAAGCTAAATATCCTGCTTTG 3´ |
| At XI-1 PAL-R | 5´ (GWR) CTCACTGAATGCAAGAAGCAAGCAGAG 3´ |
| At XI-2 PAL-F | 5´ GTGGAGGCAAAGTACCCGGCT 3´ |
| At XI-2 PAL-R | 5´ TCACTGAATGCATAGAGAAAGTAAA 3´ |
| At XI-A PAL-F | 5´ GTAGATGCTAGATATCCTGCT 3´ |
| At XI-A PAL-R | 5´ TCACTGAATGCAGGAAGAAAGCAG 3´ |
| At XI-B PAL-F | 5´ GTGGAGGCAAAATACCCGGC 3´ |
| At XI-B PAL-R | 5´ TCACTGAATGCACATAGATATTA 3´ |
| At XI-C/E PAL-F | 5´ GTTGAAGCAAAATATCCGGCT 3´ |
| At XI-C/E PAL-R | 5´ TCACTGAATGCACAATCCAAGGAG 3´ |
| At XI-D PAL-F | 5´ GTAGATGCTCGATATCCTGCTTTA 3´ |
| At XI-D PAL-R | 5´ TCACTGAATGCAGGACGAAAGCAC 3´ |
| At XI-F PAL-F | 5´ GTCGAAGCTAAATATCCGGC 3´ |
| At XI-F PAL-R | 5´ TCA ATGGATACAGAGATTTAGTAAT 3´ |
| At XI-G PAL-F | 5´ GTTGATGCCAAGGATCCAGCTT 3´ |
| At XI-G PAL-R | 5´ TCACTGGATGCAGAGAGCAAGGAC 3´ |
| At XI-H PAL-F | 5´ GTTGTGGCCAAGGATCCAGCTT 3´ |
| At XI-H PAL-R | 5´ TCACTGGATGCAAAGGGAAAGCAA 3´ |
| At XI-I PAL-F | 5´ ATAGAAGCAAGATATCCAGCA 3´ |
| At XI-I PAL | 5´ TCACTGAATGCATGATCCCAGAAG 3´ |
| At XI-K PAL-F | 5´GTTGAAGCAAAGTACCCCGCA 3´ |
| At XI-K PAL-R | 5´CTGTATACATAAGCCAAGAAGA 3´ |
| Sl XI-K PAL-F (BI930349) | 5´GTTGAGGCCAAGTATCCTG 3´ |
| Sl XI-K PAL-R (BI930349) | 5´CTGAATACATAGCCCAAGCA 3´ |
| Myo2pPAL-F | 5´AAGGAGTATGTTTCATTGGTCA 3´ |
| Myo2pPAL-R | 5´TCAGACCACAGCATTGATGG 3´ |
| GWF (attB1) | 5´ GGGGACAAGTTTGTACAAAAAAGCAGGCTTA 3´ |
| GWR (attB2) | 5´GGGGACCACTTTGTACAAGAAAGCTGGGTC 3´ |
